# Supplementary material for: The interactome of intact mitochondria by cross-linking mass spectrometry provides evidence for coexisting respiratory supercomplexes
Source: Mol Cell Proteomics. 2017 Dec 8;17(2):216–32. doi: 10.1074/mcp.RA117.000470 (PMC5795388; doi:10.1074/mcp.RA117.000470)
Supplement: Supplemental Data [file supp_RA117.000470_133922_0_supp_23978_fzffwf.zip › spectra_annotation/mito_spectra_annotation/10-1-2-1-4-1.pdf]

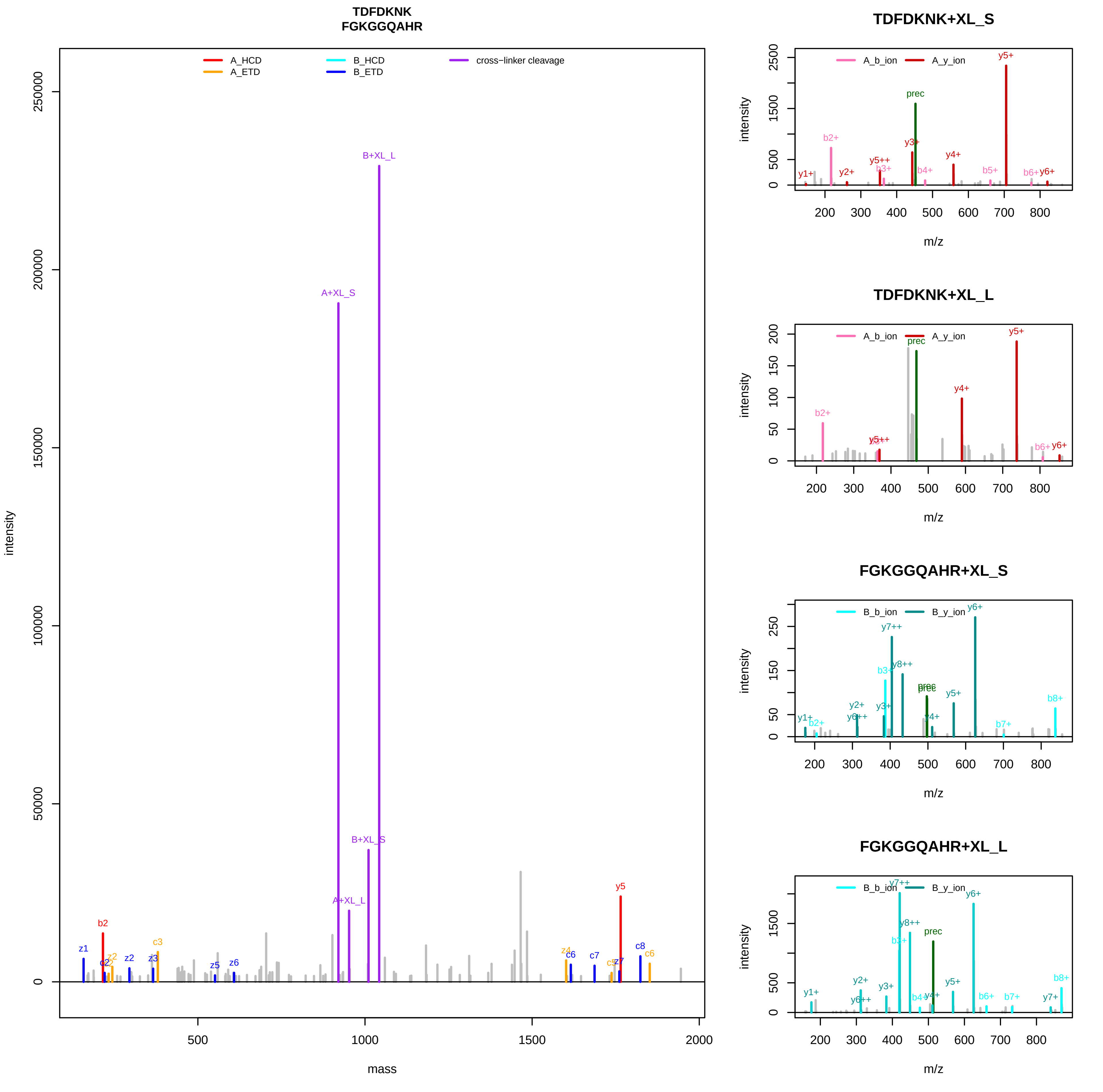

TDFDKNK
FGKGGQAHR
A_HCD
A_ETD
B_HCD
B_ETD
cross−linker cleavage
intensity
mass
B+XL_L
A+XL_S
B+XL_S
A+XL_L
y5
b2
z1
c2
z2
z3
c3
z5
z6
z4
c6
c7
c5
z7
c8
TDFDKNK+XL_S
A_b_ion
A_y_ion
prec
y1+
b2+
y2+
y5++
b3+
y3+
b4+
y4+
b5+
y5+
b6+
y6+
m/z
TDFDKNK+XL_L
FGKGGQAHR+XL_S
B_b_ion
B_y_ion
y1+
b2+
y2+
y6++
y3+
b3+
y7++
y8++
y4+
y5+
y6+
b7+
b8+
FGKGGQAHR+XL_L
b4+
b6+
y7+
